# Natural Selection in Synthetic Communities Highlights the Roles of *Methylococcaceae* and *Methylophilaceae* and Suggests Differential Roles for Alternative Methanol Dehydrogenases in Methane Consumption

**DOI:** 10.3389/fmicb.2017.02392

**Published:** 2017-12-05

**Authors:** Zheng Yu, David A. C. Beck, Ludmila Chistoserdova

**Affiliations:** ^1^Department of Chemical Engineering, University of Washington, Seattle, WA, United States; ^2^eScience Institute, University of Washington, Seattle, WA, United States

**Keywords:** synthetic community, methylotrophy, methanotrophs, *Methylobacter*, *Methylosarcina*, *Methylomonas*, *Methylophilaceae*, Lake Washington

## Abstract

We describe experiments that follow species dynamics and gene expression patterns in synthetic bacterial communities including species that compete for the single carbon substrate supplied, methane, and species unable to consume methane, which could only succeed through cooperative interactions. We demonstrate that these communities mostly select for two functional guilds, methanotrophs of the family *Methylococcaceae* and non-methanotrophic methylotrophs of the family *Methylophilaceae*, these taxonomic guilds outcompeting all other species included in the synthetic mix. The metatranscriptomics analysis uncovered that in both *Methylococcaceae* and *Methylophilaceae*, some of the most highly transcribed genes were the ones encoding methanol dehydrogenases (MDH). Remarkably, expression of alternative MDH genes (*mxaFI* versus *xoxF*), previously shown to be subjects to the rare Earth element switch, was found to depend on environmental conditions such as nitrogen source and methane and O_2_ partial pressures, and also to be species-specific. Along with the *xoxF* genes, genes encoding divergent cytochromes were highly expressed in both *Methylophilaceae* and *Methylococcaceae*, suggesting their function in methanol metabolism, likely encoding proteins serving as electron acceptors from XoxF enzymes. The research presented tested a synthetic community model that is much simplified compared to natural communities consuming methane, but more complex than the previously utilized two-species model. The performance of this model identifies prominent species for future synthetic ecology experiments and highlights both advantages of this approach and the challenges that it presents.

## Introduction

Metabolism of methane is an important part of biogeochemical cycling of carbon (Singh et al., 2010). Methane is also a major contributor to climate change (Schuur et al., 2015). A specialized group of microbes, the methanotrophs that consume methane, gaining both energy and carbon from this compound, represent a natural filter preventing an even faster escape of methane into the atmosphere (Malyan et al., 2016). While methanotrophy has been studied for the past 100 years as a metabolic feature of individual pure cultures (Kaserer, 1906; Söhngen, 1906; Trotsenko and Murrell, 2008; Chistoserdova and Lidstrom, 2013), a concept of communal function in methanotrophy has been gaining momentum (Ho et al., 2016; Yu and Chistoserdova, 2017). The mechanistic details are still scarce with regard to how and why the methanotrophs share their carbon with other species, and whether and what they gain in return (Yu and Chistoserdova, 2017). We have previously reported that feeding ^13^C-labeled methane to natural communities of Lake Washington sediment resulted in label accumulation mainly by the *Methylococcaceae* and the *Methylophilaceae* species (Kalyuzhnaya et al., 2008; Beck et al., 2013). Through microcosm manipulation, using methane as the sole source of carbon, followed by metagenomic analysis, we further confirmed that the *Methylococcaceae* and the *Methylophilaceae* were active in methane consumption (Hernandez et al., 2015; Oshkin et al., 2015). A more recent study has identified methanol as one metabolic node at which community cross-talk may be taking place (Krause et al., 2017), suggesting that methanol may be a major carbon source released by the methanotrophs, to support satellite communities. In most Gram-negative methylotrophs, methanol oxidation can be carried out by two alternative methanol dehydrogenases (MDH), the classic, MxaFI-type enzyme that has been studied for decades (Anthony, 2004; Williams et al., 2005) and the recently discovered, lanthanide (Ln^3+^)-dependent, XoxF-type MDH (Fitriyanto et al., 2011; Nakagawa et al., 2012; Pol et al., 2014). Moreover, Ln^3+^ have been implicated in a regulatory mechanism inversely controlling expression of *xoxF* and *mxaFI* genes, this mechanism known as the rare Earth element (REE) switch (Chu et al., 2016). This REE switch has been proposed to be an important factor in community function (Krause et al., 2017). Methanotrophs have also been proposed to excrete multicarbon compounds such as acetate, citrate, lactate, and succinate (Modin et al., 2007; Kalyuzhnaya et al., 2013), thus having a potential of also supporting communities of non-methylotrophic heterotrophs. A mathematical model has been recently implemented to explain carbon flow in microbial consortia consuming methane (Modin, 2017). In accordance with this model, methanotrophs feed methanol to methanol utilizers, and both methanotrophs and methanol utilizers produce organics that feed non-methylotrophic heterotrophs, in conjunction with denitrification. In accordance with the model, methanol should be available to all organisms that are capable of methanol utilization, and other organics should be available to all heterotrophs, as “public goods.” However, experimental evidence is somewhat contradictory to the notion of “public goods,” as specific species cooccurrences, such as cooperative behavior of *Methylococcaceae* and *Methylophilaceae*, have been noted not only in manipulated microcosms (Kalyuzhnaya et al., 2008; Beck et al., 2013; Hernandez et al., 2015; Oshkin et al., 2015), but also in natural populations inhabiting methane-rich environments such as permafrosts (Martineau et al., 2010; Crevecoeur et al., 2015) or methane-fueled cave biofilms (Karwautz et al., 2017). These results suggest either that many species detected in natural niches, through DNA profiling, are dormant, that some organisms may be more competitive for “public goods,” or that specific partnerships are taking place. A Black Queen Hypothesis, in accordance with which gene loss plays a role in species coevolution (Morris et al., 2012), has been evoked recently to explain non-random distribution of “public goods” among species involved in a cyanobacterial consortium, and vitamin B_12_ was proposed as an important metabolite (Lee et al., 2017). Vitamin B_12_ exchange has been previously implicated in maintaining stable cocultures of methanotrophs and non-methanotrophs (Iguchi et al., 2011).

In this manuscript, we describe experiments addressing the following questions: (1) whether the methanotrophs share carbon with non-methanotrophs as “public goods,” or whether they form specific partnerships, (2) whether all species capable of metabolizing methanol are equally competitive when present in physiologically active state, (3) whether non-methylotrophic heterotrophs also benefit from methane-derived carbon on “public goods” basis, and (4) whether transcription of any key methylotrophy genes changes in response to different environmental conditions. In these experiments, we employed synthetic communities (SCs) built of pure cultures of methanotrophs and non-methanotrophs, all previously isolated from the same ecological niche. A small subsample of these experiments has been previously published, to demonstrate the utility of SCs in basic research (Yu et al., 2016).

## Materials and Methods

### Bacterial Strains, Growth Conditions, and Experimental Setup

Bacterial strains employed in this study are listed in **Table 1** along with their characteristics relevant to methylotrophy. All have been previously isolated from Lake Washington sediment. The cultures were grown on solid media. The methanotrophs were cultivated on the nitrate minimal salts (NMS) medium in the atmosphere of methane (Dedysh and Dunfield, 2014; 25% methane, 75% air V/V). *Methylotenera mobilis* JLW8, *Methylotenera* sp. G11, and the Gram-positive methylotrophs were cultivated on the NMS medium supplemented with methylamine (0.2% W/V). The remaining methylotrophs were cultivated on the NMS medium supplemented with methanol (0.2% V/V). For the *Rhodocyclaceae*, vitamin B_12_ was added at 0.5 μg L^-1^ (Smalley et al., 2015). The non-methylotrophic heterotrophs were grown on diluted nutrient broth (BD Difco) medium (1/2 strength). Cultures were allowed to grow at room temperature (approximately 24°C) for 2–3 days, till they formed nice biofilms on the plates, after which the biomass was washed with the NMS medium, and optical density and/or cell counts were quantified, respectively, via spectrophotometry or flow cytometry (see Krause et al., 2017). The two exceptions were the *Methylobacter* strains, which were grown at 20°C, as they are psychrophilic (Kalyuzhnaya et al., 2015). The 50 strains were mixed at desired proportions (see below), and the master mixtures were incubated at 4°C for 48 h, to mimic starvation. Synthetic community 1 (SC1) master mixture was assembled by mixing the 50 strains in equal proportions, based on optical density (OD_600_). The SC2 master mixture was assembled of the same strains, as part of a separate experiment, where they were mixed based on cell counts, as determined by flow cytometry. The main difference between SC1 and SC2 was in the proportion of the methanotrophs and the *Bacillus* strains, whose cells have larger size, thus they were less abundant in SC1 compared to SC2. Incubations were carried out at 18°C in 250 mL vials, with liquid-to-headspace ratio of 1–4, as previously described (Hernandez et al., 2015; Oshkin et al., 2015). Initial inoculum OD_600_ was 0.1. Gas phase was refreshed daily according to the following scheme. (1) For the high methane high O_2_ (HH) regimen, the headspace was flushed with air for 60 s (flow rate > 1 L/min), followed by removal of 50 mL of the headspace and replacement with 50 mL of methane; (2) for the high methane low O_2_ (HL) regimen, the headspace was flushed with N_2_ for 60 s (flow rate > 1.28 L/min), followed by the removal of 60 mL of headspace and replacement with 50 mL of methane and 10 ml of air; (3) for the low methane high O_2_ (LH) regimen, the headspace was flushed with air for 60 s, followed by removal of 1 mL of headspace and replacement with 1 ml of methane; and (4) for the low methane low oxygen (LL) regimen, headspace was flushed with N_2_ for 60 s, followed by removal of 2 mL of headspace and replacement with 1 mL of methane and 1 mL of air. When cultures reached OD_600_ of approximately 0.5, they were transferred with 10-fold dilution into fresh medium. For the HH regimens, cells were transferred every 2–3 days, for the HL regimens, cells were transferred every 9–20 days, and for the LH and LL regimens, cells were transferred every 19–42 days. For the HH and HL regimens, cells from 11 time points were used for iTag profiling (Illumina sequencing of 16S rRNA gene fragment; Singer et al., 2016), for the LH and LL regimens, cells from five time points were used. For sample map and sampling schedule see **Supplementary Table S1**. Cells for DNA isolation (3 mL) were collected by centrifugation at 15,000 rpm for 5 min, and pellets were used immediately or stored at -20°C. Cells for RNA isolation (40 mL) were collected by centrifugation at 5,000 rpm for 30 min with the 1:10 volume of stop solution (5% buffer-saturated phenol in ethanol) added to cells prior to harvesting.

**Table 1:** Bacterial strains employed in the study.

Organism/strain	Phylum/class	Reference
**The methanotrophs**		
*Methylomonas* sp. LW13	Proteobacteria/Gammaproteobacteria	Kalyuzhnaya et al., 2015
*Methylomonas* sp. MK1		Kalyuzhnaya et al., 2015
*Methylomonas* sp. 11b		Kalyuzhnaya et al., 2015
*Methylobacter tundripaludum* 21/22		Kalyuzhnaya et al., 2015
*Methylobacter tundripaludum* 31/32		Kalyuzhnaya et al., 2015
*Methylosarcina lacus* LW14		Kalyuzhnaya et al., 2015
*Methylosinus* sp. PW1	Proteobacteria/Alphaproteobacteria	Beck et al., 2015
*Methylosinus* sp. LW3		Beck et al., 2015
*Methylosinus* sp. LW4		Beck et al., 2015
*Methylosinus* sp. LW5		Beck et al., 2015
		Beck et al., 2015
**The *Methylophilaceae***		
*Methylotenera mobilis* JLW8	Proteobacteria/Betaproteobacteria	Lapidus et al., 2011
*Methylotenera versatilis* 301		Lapidus et al., 2011
*Methylovorus glucosotrophus* SIP3-4		Lapidus et al., 2011
*Methylotenera mobilis* 13		Beck et al., 2014
*Methylophilaceae* 73s		Beck et al., 2014
*Methylophilaceae* 11		Beck et al., 2014
*Methylophilus methylotrophus* 1		Beck et al., 2014
*Methylophilus methylotrophus* 5		Beck et al., 2014
*Methylophilus methylotrophus* Q8		McTaggart et al., 2015b
*Methylotenera* sp. G11		McTaggart et al., 2015b
*Methylophilaceae* 7		McTaggart et al., 2015b
**Other methanol utilizers**		
*Ancylobacter* sp. 117	Proteobacteria/Alphaproteobacteria	Beck et al., 2015
*Ancylobacter* sp. 202		Beck et al., 2015
*Ancylobacter* sp. 501b		Beck et al., 2015
*Hyphomicrobium* sp. 99		Beck et al., 2015
*Hyphomicrobium* sp. 802		Beck et al., 2015
*Labrys methylaminiphilus* JLW10		Beck et al., 2015
*Methylobacterium* sp. 10		Beck et al., 2015
*Methylobacterium* sp. 77		Beck et al., 2015
*Methylobacterium* sp. 88A		Beck et al., 2015
*Methylopila* sp. 73B		Beck et al., 2015
*Methylopila* sp. 107		Beck et al., 2015
*Paracoccus* sp. N5		Beck et al., 2015
*Xanthobacter* sp. 91		Beck et al., 2015
*Xanthobacter* sp. 126		Beck et al., 2015
*Methyloversatilis discipulorum* FAM1	Proteobacteria/Betaproteobacteria	Smalley et al., 2015
*Methyloversatilis discipulorum* RZ18-153		Smalley et al., 2015
*Methyloversatilis universalis* FAM5		Smalley et al., 2015
**Non-methanol utilizing methylotrophs**		
*Aminobacter* sp. 108	Proteobacteria/Alphaproteobacteria	Beck et al., 2015
*Arthrobacter* sp. 31Y	Actinobacteria/Actinobacteria	McTaggart et al., 2015a
*Arthrobacter* sp. 35W		McTaggart et al., 2015a
*Arthrobacter* sp. MA-N2		McTaggart et al., 2015a
*Mycobacterium* sp. 141		McTaggart et al., 2015a
*Mycobacterium* sp. 155		McTaggart et al., 2015a
*Bacillus* sp. 37MA	Firmicutes/Bacilli	McTaggart et al., 2015a
*Bacillus* sp. 72		McTaggart et al., 2015a
**Non-methylotrophic heterotrophs**		
*Pseudomonas* sp. 11/12A	Proteobacteria/Gammaproteobacteria	McTaggart et al., 2015c
*Janthinobacterium* sp. RA13	Proteobacteria/Betaproteobacteria	McTaggart et al., 2015d
*Flavobacterium* sp. 83	Bacteroidetes/Flavobacteria	McTaggart et al., 2015e
*Flavobacterium* sp. Fl		McTaggart et al., 2015e


### 16S rRNA Gene Amplicon and Transcript Sequencing and Analysis

DNA was isolated using the FastDNA SPIN Kit for Soil (MP Biomedicals), according to the manufacturer’s protocol. Total RNA was isolated using the RNeasy minikit (Qiagen) as previously described (Chu et al., 2016). The purified RNA was tested for DNA contamination using 16S rRNA gene fragment PCR. Samples were stored at -80°C for subsequent analyses. iTag sequencing as well as transcript sequencing were performed at the DOE Joint Genome Institute (JGI) using standard JGI pipelines. Briefly, primers for hypervariable rRNA gene regions V4–V5 were used for iTag profiling (Parada et al., 2016). The primer sets contained Illumina linkers; a 12 bp barcode index; a pad region; a 0, 1, 2, or 3 base pair long spacer and the sequence-specific primer. Samples were pooled and run on two 2 by 300 bp Illumina MiSeq runs, essentially as described (Singer et al., 2016). RNAseq sequencing was carried out essentially as described (Beam et al., 2016).

iTag data have been archived with the IMG genome portal under JGI Project IDs 1105725 and 1105727. Transcript sequences have been archived with the IMG Genome Portal IMG genome IDs 3300009726, 3300009727, 3300009729, 3300009731, 3300009733, 3300009734, 3300009736, 3300009737, 3300009740, 3300009741, 3300009746, 3300009749, 3300009751, and 3300009752. Raw reads were aligned to the 50 respective reference genomes (see **Table 1** for reference) using the Burrows–Wheeler alignment tool version 0.7.12-r1039 (Li and Durbin, 2009), using default parameters. Raw read counts are shown in **Supplementary Table S2**. Gene expression for organisms/genes recruiting large numbers of reads were normalized manually, by calculating numbers of reads per million of total reads mapping to a specific genome and normalizing per gene length (kb).

### Bioinformatics

The UPARSE method was used for sequence processing and OTU clustering with USEARCH version 9.2.64 (Edgar, 2013). Clustering was performed at 97% sequence identity and chimeras were identified against the ChimeraSlayer reference database in the Broad Microbiome Utilities version r20110519 obtained from the UCHIME distribution (Edgar et al., 2011). Taxonomic assignments were made using the RDP Classifier from the Ribosomal Database Project version 2.12 (Wang et al., 2007). The samples were normalized so that the numbers of reads in each sample were equal.

### Phylogenetic Analysis

Protein sequences were aligned using the CLUSTAL W algorithm, and phylogenetic trees were constructed using the maximum-likelihood method, as implemented in the MEGA7 software (Kumar et al., 2016). Statistical support was obtained from 1,000 bootstrap replicates (bootstrap values >50% are reported).

## Results

### Community Dynamics in Time-Series Experiments Identify *Methylococcaceae* As the Most Competitive Methanotrophs and *Methylophilaceae* As Their Most Successful Satellite Species, under a Range of Simulated Environmental Conditions

**FIGURE 1:**
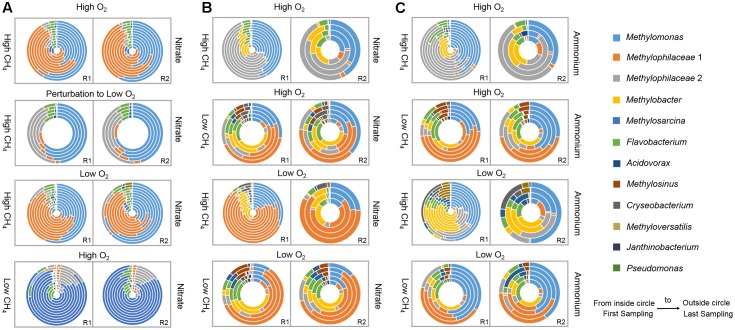
Species dynamics over time, based on iTag profiling. **(A)** SC1, incubated under three environmental conditions (two technical replicates, R1 and R2 are shown). The HH communities were perturbed by switching to HL regimen (second panel from top); **(B)** SC2, incubated under four environmental conditions, with nitrate as a nitrogen source; **(C)** SC2, incubated under four environmental conditions, with ammonium as a nitrogen source. Each circle represents community composition at a specific sampling point (5–11 time-series samples were analyzed for each microcosm). Refer to **Supplementary Table S1** for sampling schedule.

To address the questions posed, we first created SC1, by mixing 50 pure cultures of bacteria representing different functional guilds, all originating from Lake Washington sediment (**Table 1**). A single master mix was used to inoculate all vials. SC1 was incubated under three simulated environmental conditions: HH, HL, and LH (see section “Materials and Methods” and **Supplementary Table S1** for details), in two replicates, nitrate being the nitrogen source in all cases. iTag sequence profiles were generated for 11 time points for each condition, reflecting community dynamics over, respectively, 27, 141, and 212 days (**Supplementary Table S1**). Under the HH condition, communities were mostly represented by two major OTUs, *Methylophilaceae 1* (30–67% of total reads) and *Methylomonas* (12–62% of total reads), the proportion of *Methylomonas* increasing with time (**Figure 1A**). *Methylosarcina* OTU was detected in early samples (up to 23% of total reads), and the second *Methylophilaceae* OTU, *Methylophilaceae 2* was detected across samples, its relative abundance declining with time (**Figure 1A**). An OTU representing *Flavobacterium* was detected in all samples, its relative abundance declining with time. To test for response to perturbation, a switch from HH to HL at day 28 was analyzed additionally. Community response included the switch between the *Methylophilaceae* OTUs, *Methylophilaceae 2* gradually replacing *Methylophilaceae 1*, with overall increase in relative abundance of *Methylophilaceae* (**Figure 1A**).

The HL condition also selected for very simple community structure, dominated by *Methylophilaceae 1* and *Methylomonas*, with minor presence of *Methylosarcina*, *Methylophilaceae 2*, and *Flavobacterium* (**Figure 1A**). An OTU representing *Methyloversatilis* was detected in some samples at over 2% abundance. Surprisingly, an OTU representing *Acidovorax* was detected in some samples at up to 5% abundance, even though *Acidovorax* was not intentionally added to the synthetic mix. Propagation of this organism must be a result of a minor contamination present in one of the frozen culture stocks.

Species composition was dramatically different under the LH condition, with *Methylosarcina* rapidly outcompeting all other methanotroph species, and with *Methylophilaceae 2* outcompeting *Methylophilaceae 1* (**Figure 1A**). An OTU representing *Methylobacter* was detected in SC1 samples only at very low abundance. Overall, the community dynamics and trajectories were very well replicated under each regimen (**Figure 1A**).

SC2 was built independently, of the same pure cultures revived from frozen stocks. With SC2, we expanded the range of simulated conditions to include nitrogen source as a variable, substituting ammonium for nitrate, and including an LL condition, a total of eight conditions. The HH and HL regimen communities were sampled at 11 time points, while the LH and LL regimen communities were sampled at five time points (**Supplementary Table S1**).

Community dynamics under the HH regimen were similar whether nitrate or ammonium were used as nitrogen sources (**Figures 1B,C**). In both cases, the *Methylomonas* types gradually outcompeted the *Methylobacter* types, and *Methylophilaceae 2* gradually outcompeted *Methylophilaceae 1* (**Figures 1B,C**). In contrast, community dynamics appeared to be different under the HL regimen, dependent on nitrogen source. While in both conditions, *Methylomonas* gradually outcompeted *Methylobacter*, in the nitrate-supplemented medium, *Methylophilaceae* were represented almost entirely by *Methylophilaceae 1*, and the sum of *Methylococcaceae* and *Methylophilaceae* made up to over 90 and up to 97% of the entire community (**Figure 1B**). However, in the ammonium-supplemented medium, *Methylophilaceae* were represented by *Methylophilaceae 2*, and the sum of *Methylococcaceae* and *Methylophilaceae* made up to no more than 80% of the community, other relatively abundant OTUs representing *Flavobacterium*, *Methyloversatilis*, and *Acidovorax* (**Figure 1C**).

Community dynamics under low methane (LH and LL conditions) were similar to each other, independently of O_2_ concentration or nitrogen source. *Methylomonas* again outcompeted *Methylobacter*. However, in most cases, *Methylophilaceae 1* prevailed over *Methylophilaceae 2*. An exception was one replicate under the LHNH_4_ regimen, in which abundances of both *Methylophilaceae* OTUs increased with time. Under low methane, the sum of *Methylococcaceae* and *Methylophilaceae* made up approximately 80% of the total community, other abundant OTUs representing *Flavobacterium*, *Methylosinus*, and *Acidovorax*. Under the LHNO_3_ regimen, we saw a significant population of *Chryseobacterium* (up to 13%), another organism that must have originated from a minor contamination of one of the frozen stocks. Notably, *Methylosarcina* was not detected in SC2 samples. Data for technical replicates, again, agreed very well (**Figures 1B,C**).

To summarize, while alternative major players within *Methylococcaceae* and *Methylophilaceae* were involved in community dynamics, especially dramatically contrasting the “high” versus “low” methane conditions, these two guilds were the dominant species in methane consumption, corroborating our previous results (Hernandez et al., 2015; Oshkin et al., 2015). As in our previous experiments, the *Methylocystaceae* species did not appear competitive under the “high” methane regimens. They were also gradually outcompeted by *Methylococcaceae* under “low” methane regimens.

### Metatranscriptomics Provide Strain-Resolved Data on Community Composition, Identify Major Expressed Pathways, Pinpoint Alternative Methanol Dehydrogenases As Major Subjects to Regulation

**FIGURE 2:**
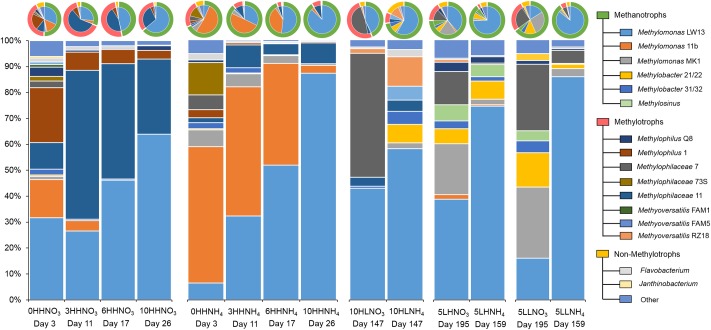
Strain-resolved transcript abundances for major expressing species, as fractions of total transcripts. For *Methylosinus*, transcripts for all four strains were combined. On top, pie charts represent the same data, along with broad-function guild abundances (outside circles). Sample designations (at the bottom) refer to number of transfers, followed by methane and O_2_ regimens (H or L), followed by the nitrogen source. The day of sampling from the beginning of the experiment is also indicated for each sample. For more information, see **Supplementary Table S1**.

Strain-resolved community compositions of SC2 microcosms were determined through transcript analysis of 14 samples (see **Supplementary Table S1**). These represented four time points for the two HH regimens (time series), and one time point for each of the remaining six regimens (**Figure 2**). Dynamics among the *Methylococcaceae* strains were dominated by *Methylomonas* strains under the HH regimens, with *Methylomonas* sp. LW13 gradually outcompeting *Methylomonas* sp. 11b and *Methylomonas* sp. MK1. However, the dynamics over time differed dependent on the source of nitrogen (**Figure 2**). Few reads were mapped to the *Methylobacter* genomes under these regimens, even though the *Methylobacter* OTU was relatively abundant at sampling points 1 and 3 in iTag analysis (**Figures 1B,C**). This suggests that *Methylobacter* species may have been transcriptionally inactive at the time of sampling. However, *Methylobacter* transcripts were detected under the HLNH_4_ and the LH and the LL regimens, in proportions predicted via the iTag analysis. *Methylosinus* transcripts were relatively abundant under the LH regimens and also under the LLNO_3_ regimen. Among the *Methylophilaceae*, *Methylophilus* species dominated over other *Methylophilaceae* under the HH regimens, *Methylophilus methylotrophus* Q8 gradually outcompeting *M. methylotrophus* 1. *Methylophilaceae* 7 was the dominant *Methylophilaceae* type under the HL and LH regimens when nitrate was supplied as the nitrogen source, and under both LL regimens. *Methyloversatilis* species outcompeted *Methylophilaceae* under the HLNH_4_ regimen. Overall, abundance of the methanotroph transcripts as a fraction of total transcripts was higher in samples supplemented with ammonium (75–95%) compared to samples supplemented with nitrate (30–75%), likely due to transcription patterns responsive to the nitrogen source, as discussed below.

**FIGURE 3:**
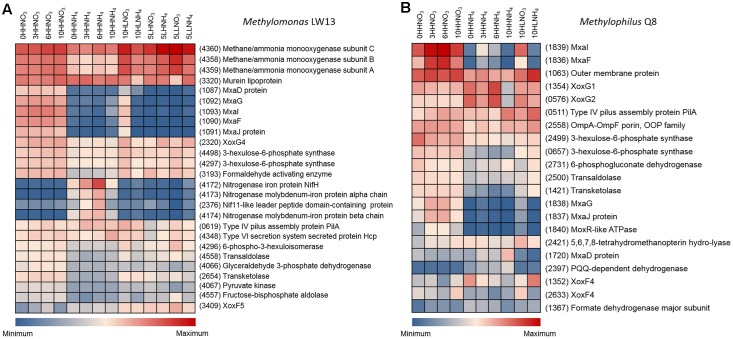
Heatmaps of some of the most highly/most differentially transcribed genes in **(A)** *Methylomonas* sp. LW13 and **(B)** *Methylophilus methylotrophus* Q8. Sample designations are as in **Figure 2**. In parentheses, protein numbers are shown as per genome annotation. See **Table 1** for reference. See Supplementary Figures S1–S4 for highly transcribed genes in other major species.

**FIGURE 4:**
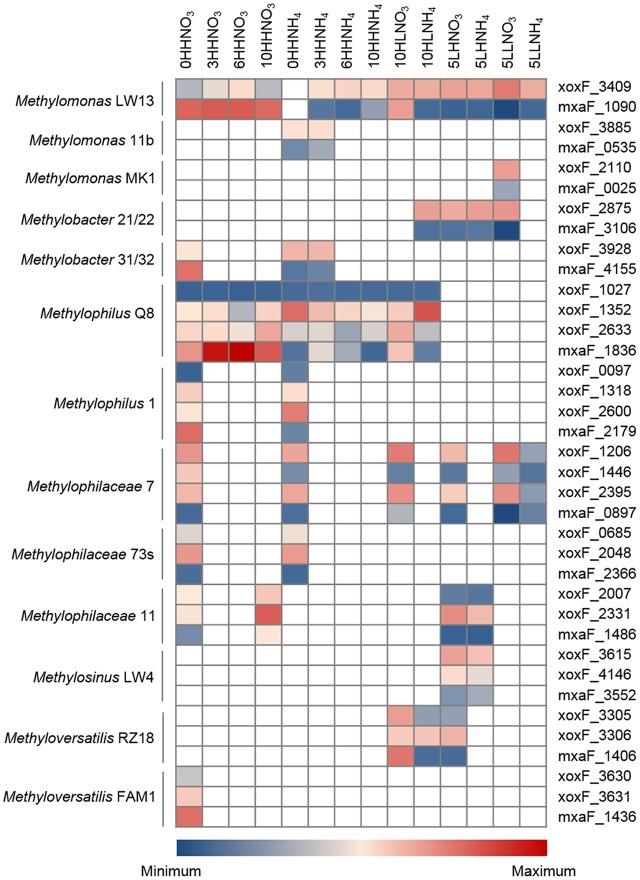
Heatmap depicting expression of *xoxF* and *mxaF* genes in major species. On the left, genus and strain names. On the right, gene name followed by gene number as per genome annotation (see **Table 1** for reference). Sample designations are as in **Figure 2**.

**FIGURE 5:**
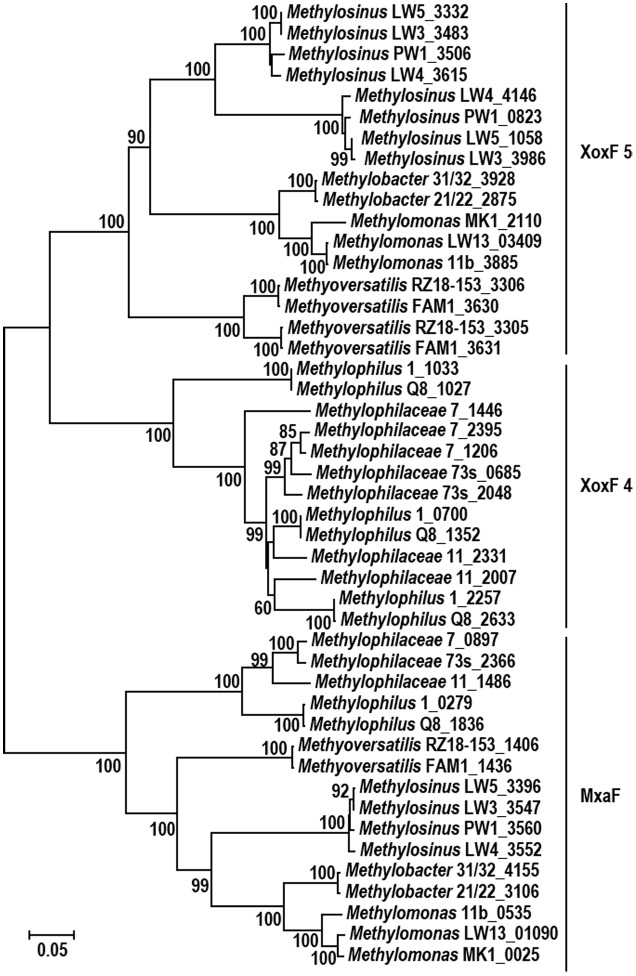
Evolutionary relationships between XoxF4, XoxF5, and MxaF proteins present in the species relevant to this study [for XoxF classification, refer to Chistoserdova (2011) and Keltjens et al. (2014)]. Genus names and strain names are shown, followed by protein numbers as per genome annotation (see **Table 1** for reference).

The most highly transcribed genes in the genomes of major species were determined through normalizing the number of transcript reads per gene length as a fraction of each species-specific transcriptome (**Figure 3**, Supplementary Figures S1–S4 and **Table S2**). Not surprisingly, genes encoding subunits of the particulate methane monooxygenase (*pmoCAB*) were most highly transcribed in the methane oxidizing species, both *Methylococcaceae* and *Methylocystaceae*, when nitrate served as the nitrogen source (**Supplementary Table S2**). Other methylotrophy functions were highly expressed in these species, as well as in the *Methylophilaceae* and the *Rhodocyclaceae* (*Methyloversatilis*). Remarkably, in all methylotrophs, some of the genes most responsive to specific experimental conditions were the genes encoding alternative MDH enzymes, MxaFI and XoxF. While the methylotroph genomes contain a single copy of each *mxaF* and *mxaI* (encoding the large and the small subunits of the Ca^2+^-dependent MDH; see **Table 1** for genome reference) the number of *xoxF* genes (encoding the single subunit of the Ln^3+^-dependent MDH) is variable. The *Methylococcaceae* possess one (Kalyuzhnaya et al., 2015), the *Methylocystaceae* and *Rhodocyclaceae* possess two (Beck et al., 2015; Smalley et al., 2015), and the *Methylophilaceae* possess up to three (Beck et al., 2014). The phylogenetic relationships of MxaF and XoxF have been reviewed previously (Chistoserdova, 2011; Keltjens et al., 2014). The expression patterns were found very distinct dependent on whether nitrate or ammonium were supplied, or whether “low” or “high” concentrations of O_2_ and methane were used (**Figure 3** and Supplementary Figures S1–S4). Moreover, expression patterns differed among different species and among different MDH enzymes. While the *Methylococcaceae*, *Methylocystaceae*, *Rhodocyclaceae*, and the *Methylophilus* species expressed *mxaFI* genes at very high levels when “high” O_2_, “high” methane, and nitrate were supplied, they expressed *xoxF* genes at higher levels when ammonium was supplied instead, or when methane was limited. Under the HLNO_3_ regimen, genes for both enzymes were expressed at approximately equal levels. In contrast, *Methylophilaceae* other than *Methylophilus* (*Methylophilaceae strains* 7, 73s, 11) displayed higher expression of *xoxF* genes under all regimens. For example, at an early sampling point under the “high” methane, “high” O_2_, nitrate, while *Methylomonas*, *Methylobacter*, *Methylophilus*, and *Methyloversatilis* were expressing genes for MxaFI at higher levels, non-*Methylophilus Methylophilaceae* species were expressing one or two genes encoding XoxF-type MDH enzymes at higher levels (**Figure 4**, Supplementary Figures S3, S4 and **Table S2**). Differential *mxaF* expression in response to the nitrogen source was confirmed on pure cultures of selected species, by real-time quantitative PCR (Supplementary Figure S5). It is noteworthy that the *xoxF* genes expressed by different community partners belong to two different phylogenetic clades (Chistoserdova, 2011), while *Methylococcaceae*, *Methylocystaceae*, and *Rhodocyclaceae* possess and express *xoxF5*, the *Methylophilaceae* possess and express *xoxF4*, and these genes are so far unique to this latter group (**Figure 5**).

**FIGURE 6:**
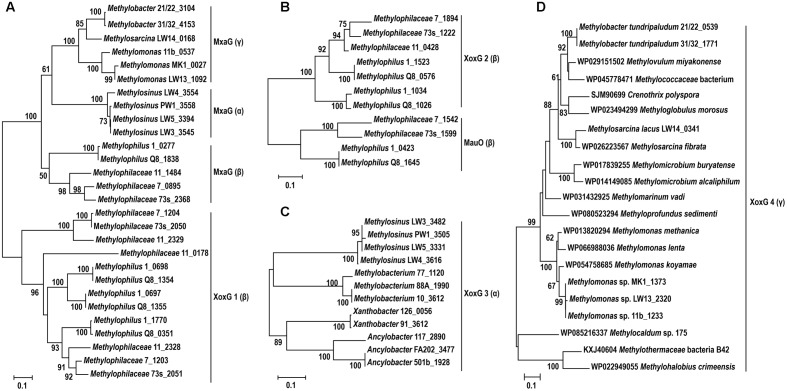
Evolutionary relationships between different XoxG proteins present in the species relevant to this study. **(A–C)** Genus names and strain names are shown, followed by protein numbers as per genome annotation (see **Table 1** for reference). **(D)** Full names are shown for organisms used in this study and for organisms whose sequences were retrieved from databases. Note that *mxaG* genes are found in α-, β-, and γ-proteobacteria (noted in parentheses after the gene name). *xoxG1* and *xoxG2* are unique to β-proteobacteria, *xoxG3* are unique to α-proteobacteria, and *xoxG4* are unique to γ-proteobacteria.

**FIGURE 7:**
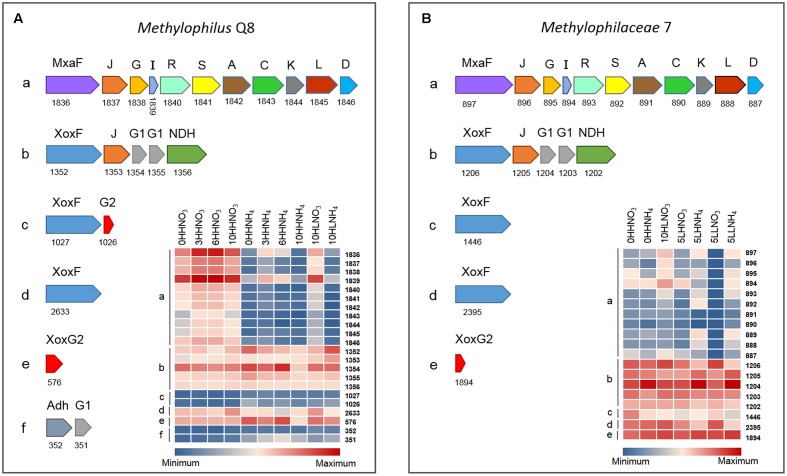
Arrangement on the chromosomes and differential expression of different *xoxF* and *xoxG* genes in different *Methylophilaceae* species **(A)** *Methylophilus* sp. Q8, **(B)** *Methylophilaceae* 7. Protein numbers are used in the heatmaps that correspond to protein numbers in gene clusters. See **Table 1** for genome reference.

In addition to the multiple genes for the MDH enzymes, genes for multiple cytochromes were highly and differentially expressed. Cytochrome MxaG, whose gene (*mxaG*) is cotranscribed with *mxaFI* genes, has been previously established as the electron acceptor from the MxaFI type MDH (Williams et al., 2006). While electron acceptors from XoxF enzymes have not been experimentally demonstrated, genes encoding cytochromes are colocalized on the chromosomes of alphaproteobacterial methylotrophs and *Methylophilaceae* (Beck et al., 2014, 2015). The arrangement is especially complex in *Methylophilaceae*, as they encode multiple cytochromes that belong to two phylogenetic lineages, XoxG1 and XoxG2, the former sharing homology with MxaG cytochromes, and the latter sharing homology with the cytochrome with a proposed function in methylamine oxidation (Kalyuzhnaya et al., 2008; **Figure 6**). In each case, genes for cytochromes representing the two phylogenetic groups were found co-expressed with specific *xoxF* genes, suggesting that they may be the natural electron acceptors from XoxF enzymes. Note that the coexpressing *xoxF* and *xoxG* genes did not always colocalize on the chromosomes (**Figure 7**).

While no cytochrome genes were present in the vicinity of the *xoxF* gene on the chromosomes of the *Methylococcaceae*, in each species we were able to identify a highly expressed gene, tentatively designated *xoxG4*, predicted to encode a novel cytochrome, with no sequence homology to either alphaproteobacterial *xoxG3* genes or the two alternative *Methylophilaceae* genes, *xoxG1* and *xoxG2* (**Figure 6**). While XoxG4 is likely an electron acceptor from XoxF5 in the *Methylococcaceae*, *xoxG4* transcription patterns differed from the ones of *xoxF5* genes (**Figure 3** and Supplementary Figure S1), suggesting that the role of this cytochrome is complex and may extend beyond methanol oxidation. Investigation of the properties of XoxG4 will be pursued in future studies.

Overall, for the *Methylococcaceae*, significant differences were noted between the transcriptomes of nitrate-supplemented versus ammonium-supplemented microcosms. In the latter, a series of hypothetical genes were expressed at very high levels, some even higher than the pMMO or the MDH genes. Interestingly, nitrogen fixation functions were expressed at high levels in ammonium-supplemented microcosms, compared to nitrate-supplemented microcosms (**Figure 3** and Supplementary Figure S1). This type of regulation was somewhat unexpected. However, it may be potentially explained by faster oxygen consumption in the ammonium-supplemented microcosms, which are characterized by higher abundance of non-methanotroph species, and thus induction of nitrogen fixation genes may be in response to hypoxia. Future research is required to test this hypothesis.

Other highly expressed genes in the *Methylococcaceae* were the ones for pilin (*pilA*), proposed to be involved in extracellular electron transfer (Holmes et al., 2017; Lovley, 2017), and for type VI secretion functions, proposed to be involved in warfare among bacterial species (Alcoforado Diniz et al., 2015; Cianfanelli et al., 2016). In the *Methylophilaceae*, *pilA* genes were also highly expressed. Multiple porin genes and genes for other outer membrane proteins were also highly expressed (**Figure 3** and Supplementary Figures S1–S4), and these may be involved in exchange of metabolites, signaling molecules, or other factors. The roles of these will also be addressed in future studies.

## Discussion

In this study, we aimed to make advances toward developing a synthetic model for studying syntrophy in aerobic methane-oxidizing communities. We employed defined SCs of methanotrophs, non-methanotrophic methylotrophs, and non-methylotrophic heterotrophs, to further test whether partnerships in aerobic methanotrophy are specific or non-specific, and also to potentially identify most prominent models for future SC-based experiments. Note that many methylotrophs included into SCs are capable of growth on a variety of organic compounds, and many are capable of denitrification (Beck et al., 2015; McTaggart et al., 2015a,e). These organisms have a potential to consume either single carbon- or multicarbon compounds, and some possess a potential to link carbon consumption to either oxygen respiration or denitrification. Thus, these organisms presented good models for testing the “public goods” hypothesis. The *Methyloversatilis* strains that require vitamin B_12_ (Smalley et al., 2015) also served as controls for vitamin B_12_ exchange potential.

All organisms were supplied into SCs in viable state, and all were presumed to be active, thus the possibility of dormancy was excluded. We assumed that members of each of the three major functional guilds would reveal competitive trends within each respective guild, and that these trends may be determined by fitness at time zero, on the one hand, and by response to specific environmental conditions, on the another hand. Thus, while we expected and indeed observed very good agreement in terms of community dynamics between the technical replicates (**Figures 1A–C**), good replication between independent experiments was not necessarily expected. In this sense, the SC approach presents some serious methodological challenges. First, as the organisms involved can survive and thrive on their own, as pure cultures, the choice of experimental conditions conducive of cooperative behavior may be important (medium composition, partial pressures of methane and O_2_, nitrogen sources, etc.). Second, the relative fitness at time zero is hard to control. For example, it is not clear on what substrate the pure cultures should be pregrown, to better reflect their diet *in situ*. This problem is especially profound when it comes to organisms with broad metabolic capabilities. Assuming that most organisms must be limited by some nutrient in their natural habitat (be it O_2_, carbon, nitrogen, or an essential metal), we elected to starve the premixed SCs, incubating them in a minimal medium, before supplying them with the carbon source. However, different organisms may respond differently to this mimicked “starvation,” and thus initial fitness of different organisms would depend on their stress response mechanisms and their robustness. Additionally, other factors may be important that may control species ratios in natural communities, including functional guilds not primarily involved in methane utilization or cometabolism, but sporadically influencing community structure, for example, predatory species, species harboring predatory plasmids or phages, or free-living phages.

Despite some differences between the dynamics among the specific strains of *Methylococcaceae* and *Methylophilaceae* in SC1 versus SC2, general trends at the level of functional guilds remained the same. In each case, *Methylococcaceae* and *Methylophilaceae* became the dominant species, quickly outcompeting *Methylocystaceae* as well as other methylotrophic and non-methylotrophic heterotrophs. In most cases, the *Methylomonas* species became dominant. This would be predicted from their growth characteristics in pure cultures, in standard conditions, under which they outperform *Methylobacter*, *Methylosarcina*, or *Methylosinus* (Auman et al., 2000 and unpublished results). However, *Methylomonas* species have not been detected as dominant in prior experiments that involved natural sediment samples (Kalyuzhnaya et al., 2008; Beck et al., 2013; Hernandez et al., 2015; Oshkin et al., 2015), possibly due to existence of some specific controls (predation, antibiotic regulation, molecular signaling) that were not recreated in SCs in this study.

The trend of the *Methylophilaceae* outcompeting other methanol utilizers, noted for natural communities (Hernandez et al., 2015; Oshkin et al., 2015), persisted in SCs. While many species included into SCs show robust growth on methanol in laboratory (e.g., *Methylobacterium*, *Paracoccus*, *Hyphomicrobium*, *Methylopila*; Beck et al., 2015), these species did not seem competitive under most regimens. As an exception, *Methyloversatilis* species were prominent under the “low” methane regimens in SC2. These observations suggest that the *Methylophilacaea*, and, under some conditions, *Methyloversatilis* species have some advantages over alphaproteobacterial methylotrophs in consuming methanol. Success of the latter also suggests that vitamin B_12_ indeed could be shared among the community members.

The heterotrophs that could potentially consume a variety of organic compounds, i.e., the Gram-positive species and the facultatively methylotrophic alphaproteobacteria, did not persist in the microcosms, in agreement with prior results with “natural” microcosms (Hernandez et al., 2015; Oshkin et al., 2015). In contrast, *Pseudomonas* and *Janthinobacterium* were detected in some samples at over 2% abundance, suggesting that they were more competitive. However, *Flavobacterium* species, and also “uninvited,” contaminants belonging to *Chryseobacterium* (which are also Bacteroidetes) and *Acidovorax* (Burkholderiales, closely related to *Janthinobacterium*) were measured at even higher abundances, suggesting that Bacteroidetes and *Acidovorax*/other Burkholderiales must have strong competitive advantages over other, methylotrophic or non-methylotrophic heterotrophs. Overall, our data continue to suggest that the carbon released by the methanotrophs is not equally accessible by all organisms present in the community, and thus some mechanisms must exist that make *Methylophilaceae* and, under certain conditions, *Rhodocyclaceae* more competitive for methanol, *Acidovorax* more competitive for acetate and/ other organic acids, while *Bacteroidetes* appear more competitive in consumption of polymeric substances that are excreted by other species.

Highly covered transcriptomes were obtained for major partner species. Remarkably, among the most highly and most differentially expressed were the genes for alternative MDH enzymes, MxaFI, the classic Ca^2+^-dependent MDH and XoxF, the recently discovered MDH requiring REEs (Chistoserdova, 2016; Martinez-Gomez et al., 2016). These enzymes have been previously found to be subjects of the so-called REE switch (Chu et al., 2016), a mechanism that inversely regulates transcription from *xox* and the *mxa* gene clusters in response to the presence of REEs (Chu and Lidstrom, 2016; Gu et al., 2016; Vu et al., 2016). However, the straightforward nature of the switch has already been questioned. For example, in an alphaproteobacterial methanotroph *Methylosinus trichosporium* OB3b, copper appears to override the switch (Gu et al., 2016; Gu and Semrau, 2017). The switch also appears to work differently dependent on whether methanotrophs are cultivated as pure cultures or as members of communities (Krause et al., 2017). Thus, further insights are required to better understand the mechanism of the REE switch.

The data we present here indicate that the REE switch must be a subject to much more complex regulation than previously appreciated. (1) First, we demonstrate that the REE switch is responsive to a nitrogen source. As our experimental design did not include added REEs, high expression of the *mxa* genes was expected. However, this was true only for the HHNO_3_ regimen, the standard laboratory condition (Chu and Lidstrom, 2016). Even under this regimen, *Methylophilaceae* other than *Methylophilus* tended to overexpress *xoxF* over *mxaF*. (2) Second, we demonstrate that both O_2_ and methane partial pressures also have control over the REE switch, high methane selecting for the MxaFI–MDH (under nitrate), “low” methane and “low” O_2_ selecting for XoxF, and “high” methane “low” O_2_, nitrate allowing for transcription of both systems at similar levels. (3) Third, we demonstrate that, when multiple copies of *xoxF* genes are present in a single genome, they are not all following the same regulation pattern. (4) Finally, different organisms operate the RRE switch differently, such as in the samples where *Methylophilus* types express the *mxa* genes at a higher level, other *Methylophilaceae* preferentially express *xoxF* genes.

Besides multiple *xoxF* genes, multiple cytochrome-encoding genes (*xoxG*) were also highly and differentially expressed, adding further complexity to the REE switch. Moreover, while *Methylophilaceae* tended to coexpress *xoxF* and *xoxG* genes, expression of *xoxG* in *Methylococcaceae* was not coordinated with expression of *xoxF*. While the meaning of such complexity for the REE switch remains enigmatic, it further points to the importance of the methanol oxidation step in communal metabolism of methane and warrants further investigations.

## Conclusion

We here demonstrated the utility of multispecies SCs in studying complex biogeochemical processes, such as communal metabolism of methane, in a simplified, controllable model. We demonstrate that general trends in SCs mimic the ones in natural communities, selecting for *Methylococcaceae* and *Methylophilaceae* under most conditions, thus defying the hypothesis of random distribution of “public goods.” Through metatranscriptome analysis, we uncover the unexpected complexity of transcriptional regulation of methanol oxidation, mediated through the REE switch, which, in turn, is governed by multiple environmental factors. We also demonstrate that the REE switch acts differently in different organisms and on different XoxF/XoxG enzymes. These new data will inform future development of SCs customized toward specific experimental goals, in order to target specific functions that may contribute to cooperative behavior in methane consumption.

## Author Contributions

ZY and LC conceived the study, ZY carried out experiments, DB carried out bioinformatics analyses, and ZY and LC analyzed the data and wrote the manuscript.

## Conflict of Interest Statement

The authors declare that the research was conducted in the absence of any commercial or financial relationships that could be construed as a potential conflict of interest.

## Supplementary Material

TABLE S1 Sample map.

TABLE S2 Raw read counts.
