# Supplementary material for: Natural Selection in Synthetic Communities Highlights the Roles of Methylococcaceae and Methylophilaceae and Suggests Differential Roles for Alternative Methanol Dehydrogenases in Methane Consumption
Source: Front Microbiol. 2017 Dec 5;8:2392. doi: 10.3389/fmicb.2017.02392 (PMC5723320; doi:10.3389/fmicb.2017.02392)
Supplement: Supplementary file 3 [file Presentation_1.PDF]

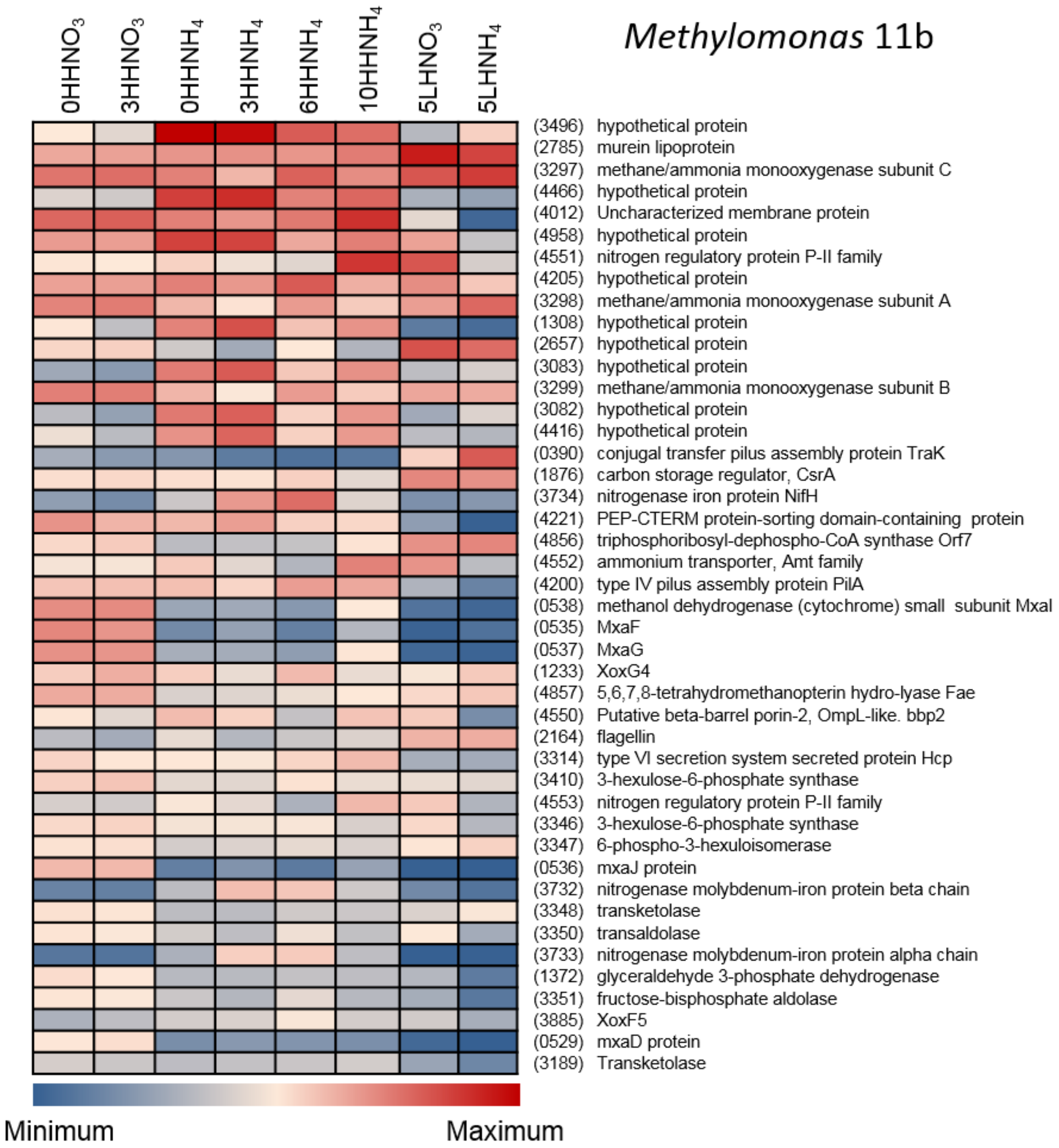

Figure S1. Heatmap of some of the most highly transcribed genes in *Methylomonas* sp. 11b.

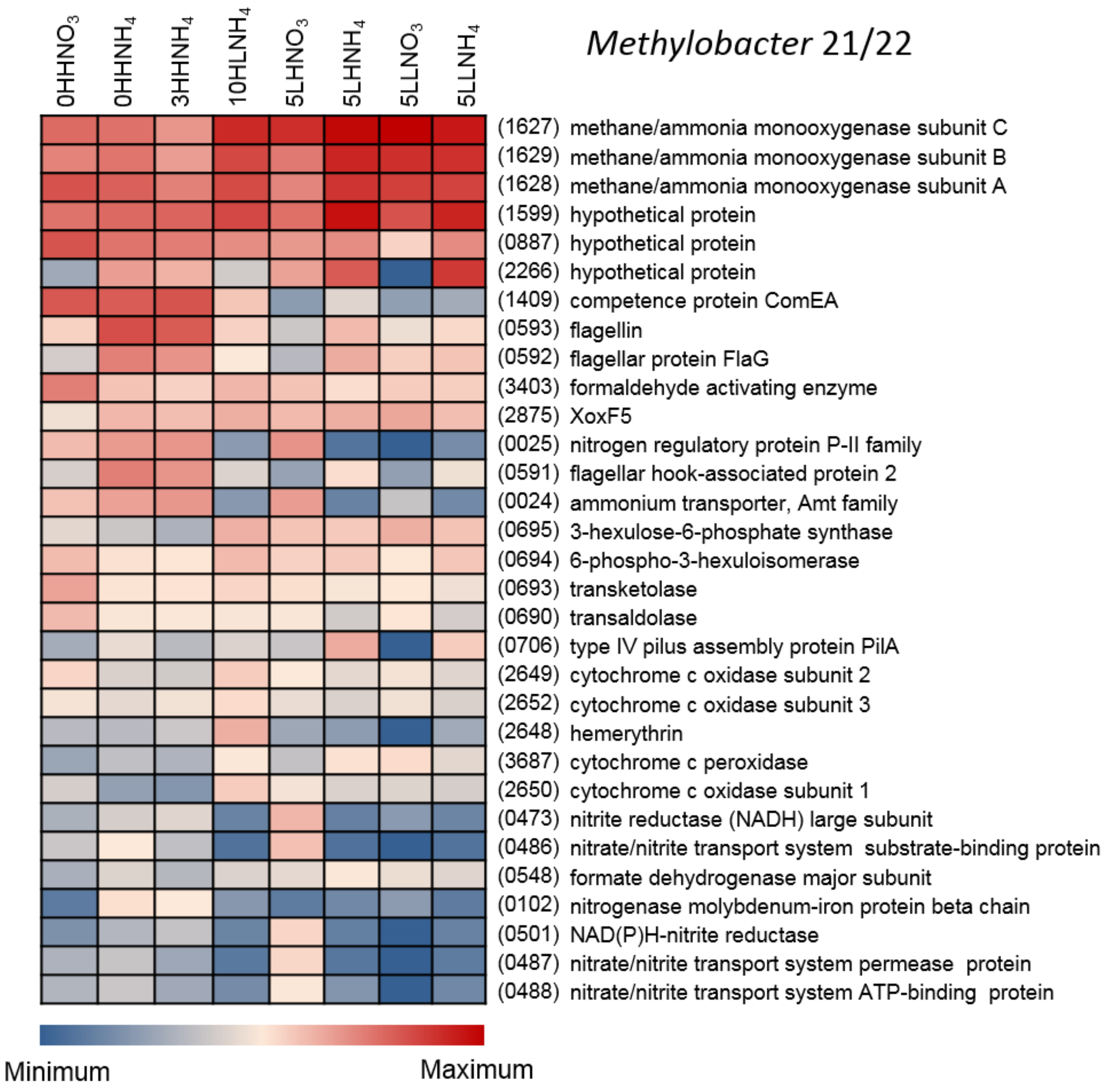

Figure S2. Heatmap of some of the most highly transcribed genes in *Methylobacter tundripaludum* 21/22.

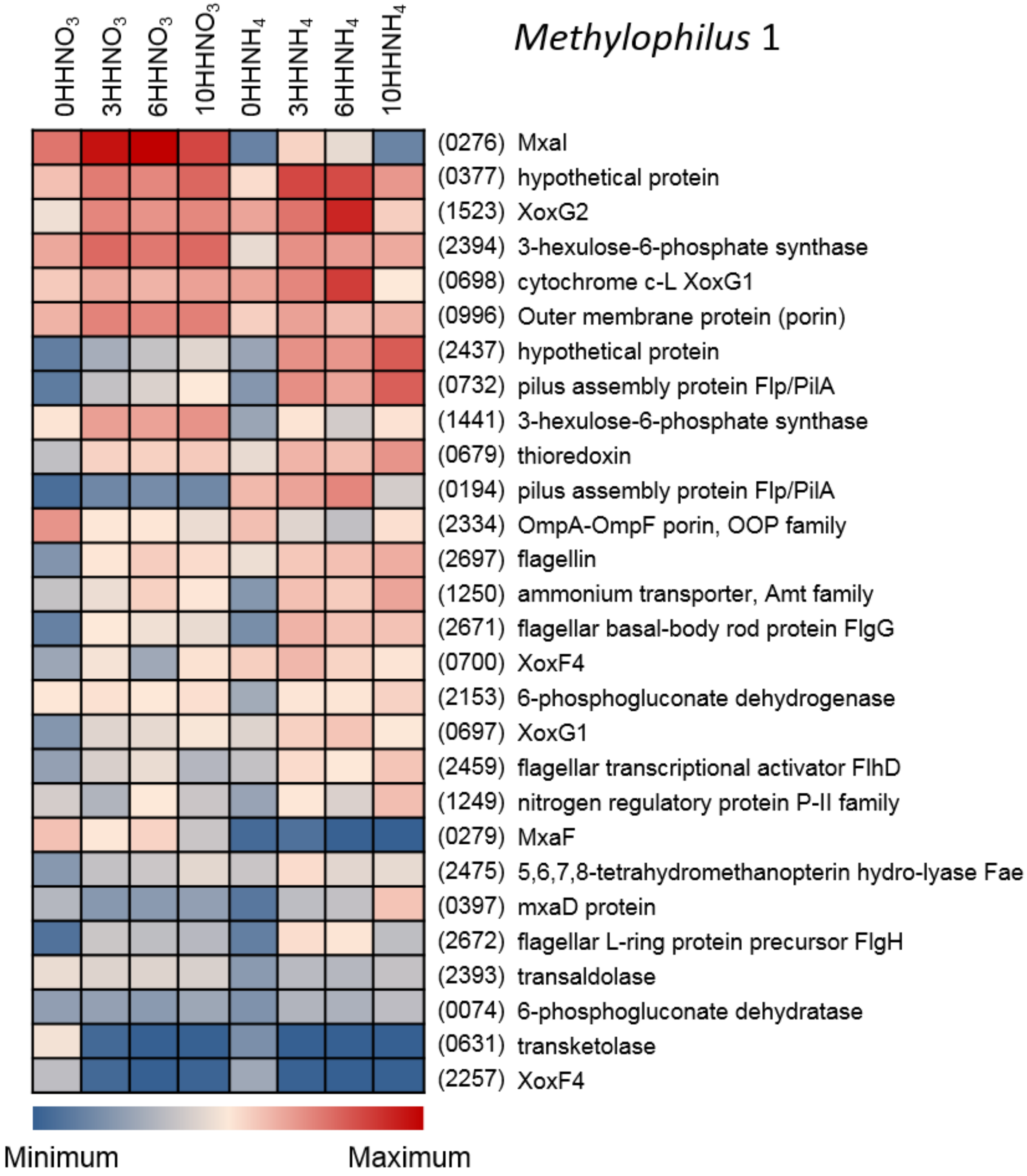

Figure S3. Heatmap of some of the most highly transcribed genes in *Methylophilus methylotrophus* 1.

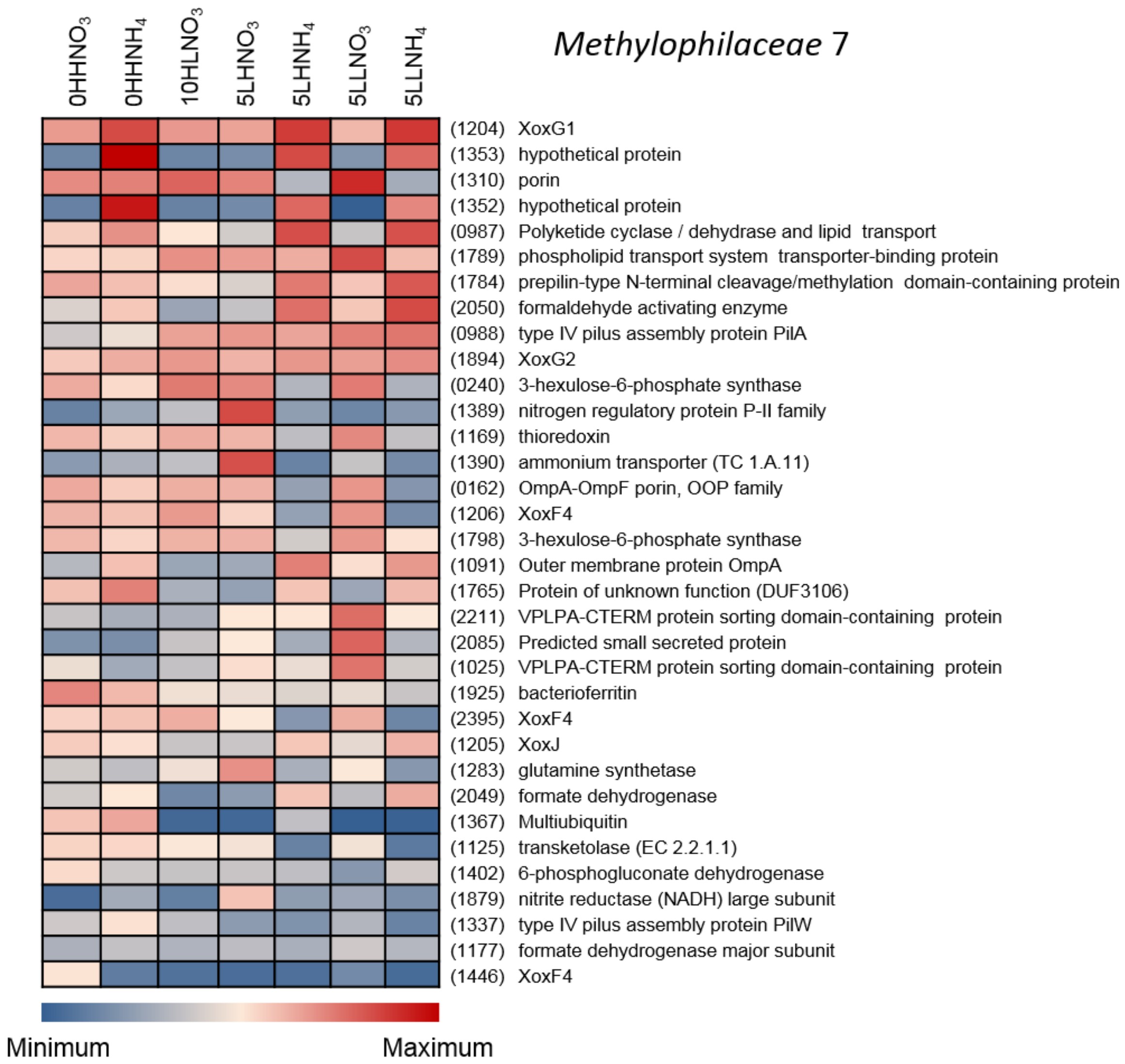

Figure S4. Heatmap of some of the most highly transcribed genes in *Methylophilaceae* 7.

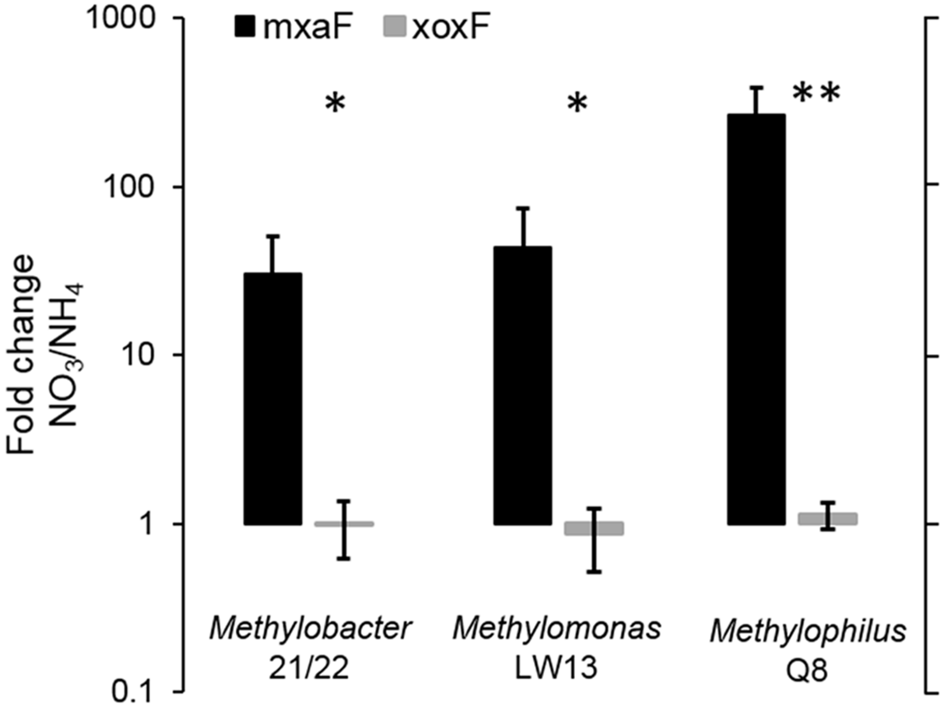

Figure S5. qRT-PCR values depicting the fold change in expression of *mxaF/xoxF* gene pairs in selected pure cultures grown with nitrate or ammonium as nitrogen sources. A t-test was performed to determine the significance of changes in gene-expression levels: $**P < 0.01$, $*P < 0.05$. Error bars indicate the SE (n = 3). The methanotrophs were grown on methane (HH regimen), *Methylophilus methylotrophus* Q8 was grown on methanol (0.1% V/V). Cells were harvested by centrifugation when the OD_{600} reached approximately 0.5. 1:10 volume of stop solution (5% buffer-saturated phenol in ethanol) was added to cells prior to cell harvesting. Cells were resuspended in RNA extraction buffer (1:3 ratio of 5% cetrimonium bromide in 2.5 M NaCl to 0.1 M phosphate buffer [pH 5.8]) and lysed by bead beating with FastPrep Lysing Matrix E tubes (MP Biomedicals) in 50% phenol–chloroform–isoamyl alcohol (at a 25:24:1 ratio), 0.5% sodium dodecyl sulfate, and 0.5% *N*-lauroylsarcosine sodium salt. After centrifugation, the aqueous layer was harvested and mixed with an equal volume of chloroform-isoamyl alcohol (24:1 ratio). The aqueous layer was harvested again, and RNA was precipitated with 150 mM sodium acetate, 1.5 mM $MgCl_2$, and 50% isopropanol (all final concentrations) overnight at −80°C. The precipitated RNA was harvested by centrifugation and treated with DNase I (Life Technologies) before purification with the RNeasy minikit and RNase-free DNase

(Qiagen). The purified RNA was tested for DNA contamination using 16S rRNA gene PCR amplification test. cDNA was generated using ~500 ng of RNA as the template with the SensiFast cDNA synthesis kit (Bioline). PCR mixtures consisted of the following: 400 µM gene-specific primers, SensiFast SYBR No-Rox kit (Bioline), cDNA, and double-distilled H_2O up to 10 µl of volume. The PCR mixtures were placed into LightCycler capillaries (Roche Diagnostics), and reactions were run using a LightCycler 2.0 (Roche Diagnostics). Threshold cycle (C_T) values were determined using LightCycler software, version 3.5 (Roche), and all gene expression values were normalized to 16S rRNA C_T values. For *M. methylotrophus*
